# Prehospital transportation of severe penetrating trauma victims in Sweden during the past decade: a police business?

**DOI:** 10.1186/s13049-023-01112-x

**Published:** 2023-09-08

**Authors:** Mattias Renberg, Martin Dahlberg, Mikael Gellerfors, Amir Rostami, Mattias Günther, Elham Rostami

**Affiliations:** 1 Department of Anesthesiology and Intensive Care, Södersjukhuset, Stockholm, Sweden; 2 Department of Surgery, Södersjukhuset, Karolinska Institutet, Stockholm, Sweden; 3 Department of Perioperative Medicine and Intensive Care, Karolinska University Hospital, Stockholm, Sweden; 4 Rapid Response Car, Capio, Stockholm, Sweden; 5 Department of Physiology and Pharmacology, Karolinska Institutet, Stockholm, Sweden; 6 Swedish Air Ambulance (SLA), Mora, Sweden; 7 Department for Social Work and Criminology, University of Gävle, Gävle, Sweden; 8 Experimental Traumatology Unit, Department of Neuroscience, Karolinska Institutet, Stockholm, Sweden; 9 Department of Clinical Science and Education, Section for Anesthesiology and Intensive Care, Södersjukhuset, Karolinska Institutet, Sjukhusbacken 10, S1, 118 83 Stockholm, Sweden; 10 Department of Medical Sciences, Section of Neurosurgery, Uppsala University Hospital , Uppsala, Sweden

**Keywords:** Penetrating trauma, Trauma, Police, Prehospital, Private vehicle

## Abstract

**Introduction:**

Sweden is facing a surge of gun violence that mandates optimized prehospital transport approaches, and a survey of current practice is fundamental for such optimization. Management of severe, penetrating trauma is time sensitive, and there may be a survival benefit in limiting prehospital interventions. An important aspect is unregulated transportation by police or private vehicles to the hospital, which may decrease time but may also be associated with adverse outcomes. It is not known whether transport of patients with penetrating trauma occurs outside the emergency medical services (EMS) in Sweden and whether it affects outcome.

**Method:**

This was a retrospective, descriptive nationwide study of all patients with penetrating trauma and injury severity scores (ISSs) ≥ 15 registered in the Swedish national trauma registry (SweTrau) between June 13, 2011, and December 31, 2019. We hypothesized that transport by police and private vehicles occurred and that it affected mortality.

**Result:**

A total of 657 patients were included. EMS transported 612 patients (93.2%), police 10 patients (1.5%), and private vehicles 27 patients (4.1%). Gunshot wounds (GSWs) were more common in police transport, 80% (n = 8), compared with private vehicles, 59% (n = 16), and EMS, 32% (n = 198). The Glasgow coma scale score (GCS) in the emergency department (ED) was lower for patients transported by police, 11.5 (interquartile range [IQR] 3, 15), in relation to EMS, 15 (IQR 14, 15) and private vehicles 15 (IQR 12.5, 15). The 30-day mortality for EMS was 30% (n = 184), 50% (n = 5) for police transport, and 22% (n = 6) for private vehicles. Transport by private vehicle, odds ratio (OR) 0.65, (confidence interval [CI] 0.24, 1.55, p = 0.4) and police OR 2.28 (CI 0.63, 8.3, p = 0.2) were not associated with increased mortality in relation to EMS.

**Conclusion:**

Non-EMS transports did occur, however with a low incidence and did not affect mortality. GSWs were more common in police transport, and victims had lower GCS scorescores when arriving at the ED, which warrants further investigations of the operational management of shooting victims in Sweden.

## Introduction

Gun homicide increased in Sweden during the past decade, in contrast to a decreasing incidence in the majority of European countries [1]. Transitioning from a society with relatively few shootings, first responders on scene now face a new reality. It is known that the outcome of severe penetrating trauma is time sensitive [2–4]. There may be a survival benefit from limiting prehospital interventions in severely injured patients in favor of urgent transport in urban settings, although optimal prehospital management is debated [5–9]. Some of the deaths may be preventable depending on prehospital care, and “scoop and run” may be preferable to “stay and play” in select cases [10, 11]. The ultimate “scoop and run” approach is immediate transport of the victim to the hospital by police. The police are often first on scene, which may decrease the time from injury to arrival at definitive care.[12–15] However, these transports provide only a bare minimum of medical intervention. The first organized police transport approach was established in Philadelphia in 1996 [12]. By 2016, more than 50% of the penetrating trauma in Philadelphia was transported by police to medical facilities [16]. These patients presented with a higher injury severity score (ISS), lower Glasgow coma scale score (GCS), and a higher frequency of gunshot wounds (GSWs) than those transported by EMS [8, 16–19], and it is still debated whether a survival benefit can be deducted. Initial reports showed an increase in mortality for police transport compared with emergency medical services (EMS), although adjusted comparisons indicated no difference [8, 16–19] and one report indicated a survival benefit [15]. The picture was further complicated by the fact that transport by private vehicles decreased the adjusted mortality in relation to EMS [20]. It is not known whether transport of patients with penetrating trauma occurs outside of the EMS in Sweden or whether it affects outcome. Sweden is a relatively large country compared to its population, and level one trauma centers are located only in urban areas [21]. Therefore, data from the US cannot be extrapolated to Sweden. Moreover, prehospital care cannot be compared directly, as organizations, operative competence, and mandates differ substantially between countries [22–24]. Sweden is facing a surge of gun violence that mandates optimized prehospital transport approaches, and an understanding of current practice is fundamental for such optimization. Therefore, we used the Swedish National Trauma Registry (SweTrau) to investigate prehospital transportation modalities of severe, penetrating trauma in Sweden during 2011–2019. We hypothesized that transport by police and private vehicles occurred and that it affected mortality.

## Methods

### Study population

This was a retrospective, descriptive nationwide study of all patients with penetrating trauma and ISS ≥ 15 registered in SweTrau between its establishment on June 13, 2011, and December 31, 2019. The population in Sweden was 9,415,570 people in 2011 and 10,327,589 people in 2019. Patients of all ages and sexes were included. The study was approved by the Swedish Ethical Review Authority (no 2019–02842) and by the SweTrau steering group.

### Swedish trauma registry

Data were extracted from the national trauma registry in Sweden, SweTrau, which was established in 2011. In 2019, 92% of all hospitals in Sweden with trauma capabilities (anesthesia, surgery and radiology competence available at all times) were associated with SweTrau, and 86% of hospitals in the registry reported actively [25]. SweTrau follows “the revised Utstein Trauma Template for Uniform Reporting of Data following Major Trauma, 2009, a uniform template for reporting variables and outcomes in trauma allowing comparison of trauma systems in Europe [26]. SweTrau estimates its coverage by comparing registry entries of trauma requiring intensive care with data in The Swedish Intensive Care registry (SIR) of admissions with the diagnosis “Trauma” and injury diagnoses SA01-TA04 and TA09-TA13. SweTrau’s coverage was estimated at 72.6% in 2019 [25]. To be included in SweTrau, patients needed to fulfill at least one of the following criteria: exposure to a traumatic event with subsequent trauma team activation at the receiving hospital, ISS > 15 without trauma team activation, ISS > 15 and transferred to a participating hospital within 7 days of the trauma. The exclusion criteria for registration in SweTrau were trauma team activation without a precipitating trauma and patients where the only injury was a chronic subdural hematoma.

### Definitions and missing data

Penetrating trauma was defined as injuries caused by sharp objects. Transport by EMS was defined as ground ambulance. Airborne EMS and transports between hospitals were excluded. Scene time was defined as the registered time from EMS arrival to the scene of trauma until departure, and transport time was defined as the registered time from EMS departure from the scene of trauma to arrival at the receiving hospital. Prehospital time was defined as scene time combined with transport time. Missing data are presented with their respective categories in tables when applicable. Patients arriving on foot were excluded from Tables 2 and 3 due to isolated patients.

### Statistical analyses

Data are presented as mean with interquartile range (IQR) for continuous variables. Descriptive statistics of patient characteristics are presented as numbers and percentages. Data analysis was performed with R (v. 4.0.3). Logistic regression models for dichotomous outcomes were used with restricted cubic splines and three knots placed at their respective quantiles. P < 0.05 was considered statistically significant.

## Results

**Fig. 1:**
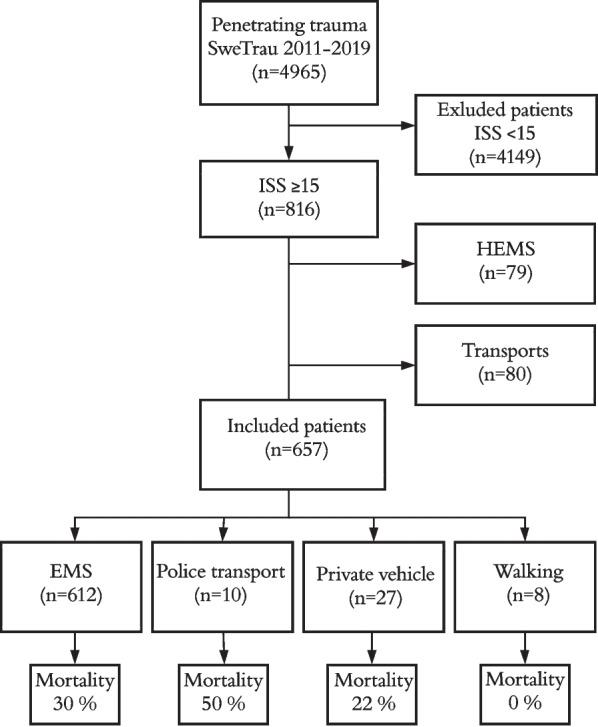
Flowchart of patient inclusion. *EMS* emergency medical service, *HEMS *helicopter emergency medical service, *ISS* injury severity score, *SweTrau* Swedish national trauma registry

**Fig. 2:**
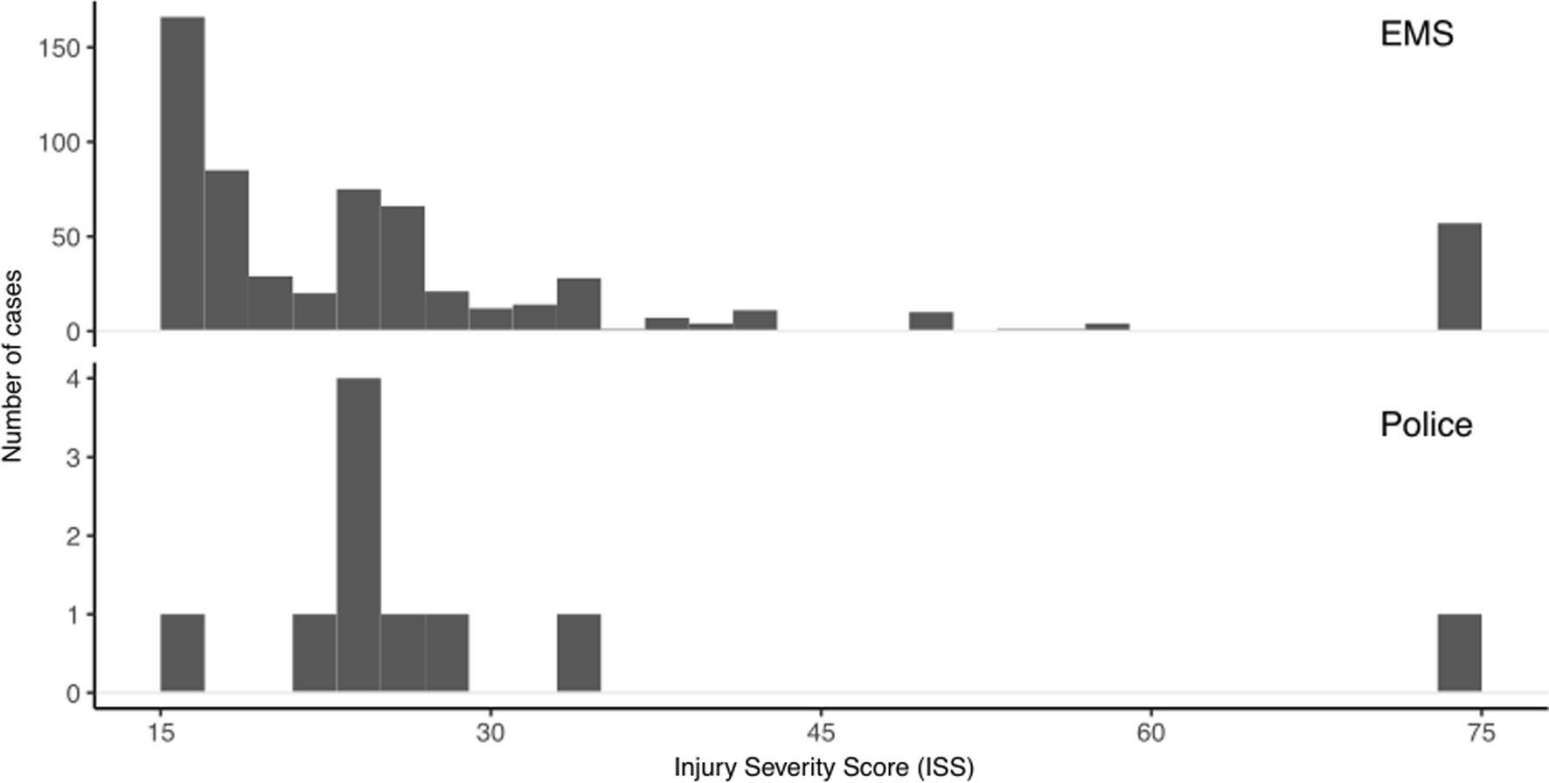
Histogram visualizing ISS in patients transported by EMS and police. The police transported patients with lower ISS to a lesser extent compared with EMS. *EMS *emergency medical service, *ISS *injury severity scale

In total, 657 patients were included in the study (Fig. 1). The EMS transported 612 patients (93.2%), the police transported 10 patients (1.5%), private vehicle transported 27 patients (4.1%), and 8 patients (1.2%) arrived at the emergency department (ED) by foot (Table 1). The median age was 30 years (IQR 22, 45) for patients transported by EMS, 23 (IQR 20.5, 29) for police transport and 26 (IQR 22, 28.5) for private vehicles. The median ISS was 25 in patients transported by both EMS (IQR 17, 29) and police (IQR 24.25, 28.25) and 20 (IQR 16, 34.5) for private vehicles. A histogram visualizing ISS for patients transported by EMS and police is displayed in Fig. 2. GSW was more common in patients transported by police (80%, n = 8) than in those transported by private vehicles (59%, n = 16) and EMS (32%, n = 198). The GCS score in the ED was lower for patients transported by police, 11.5 (IQR 3, 15), in relation to EMS, 15 (IQR 14, 15) and private vehicles 15 (IQR 12.5, 15). The median blood pressure was 110 mmHg (IQR 40, 146) for patients with police transport, 120 mmHg (IQR 90, 140) for EMS and 124 mmHg (IQR 94, 139) for private vehicles. The median scene time for EMS was 12 min (IQR 8, 19), and the median transit time was 12 min (IQR 7, 19). Patients’ injuries are presented in Table 2.

**Table 1:** Baseline characteristics

Characteristic	EMS, N = 612^a^	Police, N = 10^a^	Private vehicle, N = 27^a^	Walking, N = 8^a^
Age (years)	30 (22,45)	23 (20.5, 29)	26 (22, 28.5)	25.5 (23,29)
(Missing)	1	0	0	0
*Sex*				
Female	62/612 (10%)	0/10 (0%)	1/27 (3.7%)	0/8 (0%)
Male	550/612 (90%)	10/10 (100%)	26/27 (96%)	8/8 (100%)
Unknown	0/612 (0%)	0/10 (0%)	0/27 (0%)	0/8 (0%)
Injury severity score	25 (17, 29)	25 (24.25, 28.25)	20 (16, 34.5)	18 (17, 19.5)
*Injury mechanism*				
GSW	198/612 (32%)	8/10 (80%)	16/27 (59%)	2/8 (25%)
SW	369/612 (60%)	2/10 (20%)	11/27 (41%)	5/8 (62%)
Other	45/612 (7.4%)	0/10 (0%)	0/27 (0%)	1/8 (12%)
ED GCS score	15 (14, 15)	11.5 (3, 15)	15 (12.5, 15)	15 (14.75, 15)
First ED blood pressure (mmHg)	120 (90, 140)	110 (40, 146)	124 (94, 139)	144.5 (131.25, 160.25)
(Missing)	118	3	6	0
*First ED blood pressure (RTS)*				
No carotid	71/101 (70%)	1/2 (50%)	5/6 (83%)	0/0 (NA%)
Only carotid	8/101 (7.9%)	1/2 (50%)	0/6 (0%)	0/0 (NA%)
Femoral	4/101 (4.0%)	0/2 (0%)	1/6 (17%)	0/0 (NA%)
Weak radial	7/101 (6.9%)	0/2 (0%)	0/6 (0%)	0/0 (NA%)
Clear radial	11/101 (11%)	0/2 (0%)	0/6 (0%)	0/0 (NA%)
(Missing)	494	7	21	8
Time spent at scene (mins)	12 (8, 19)	NA (NA, NA)	NA (NA, NA)	NA (NA, NA)
(Missing)	0	10	24	8
Time spent in transit from scene to hospital (mins)	12 (7, 19)	NA (NA, NA)	NA (NA, NA)	NA (NA, NA)
(Missing)	4	10	27	8
*First respiratory rate (/min)*				
0	56/426 (13%)	3/9 (33%)	5/21 (24%)	0/8 (0%)
1–9	3/426 (0.7%)	0/9 (0%)	0/21 (0%)	0/8 (0%)
10–29	307/426 (72%)	6/9 (67%)	15/21 (71%)	8/8 (100%)
> 29	60/426 (14%)	0/9 (0%)	1/21 (4.8%)	0/8 (0%)
(Missing)	186	1	6	0

^a^Median (IQR); n/N (%)

*ED* emergency department, *EMS* emergency medical service, *GCS* Glasgow coma scale, *GSWs* gunshot wounds, *SWs* stab wounds

**Table 2:** Patient injuries

Characteristic	EMS, N = 612^a^	Police, N = 10^a^	Private vehicle, N = 27^a^
Head	167 (27%)	3 (30%)	4 (15%)
Face	145 (24%)	2 (20%)	5 (19%)
Neck	95 (16%)	0 (0%)	3 (11%)
Thorax	406 (66%)	5 (50%)	17 (63%)
Abdomen	253 (41%)	4 (40%)	14 (52%)
Spine	84 (14%)	2 (20%)	3 (11%)
Upper extremity	245 (40%)	1 (10%)	9 (33%)
Lower extremity	194 (32%)	3 (30%)	10 (37%)

^a^Median (IQR); n/N (%). *EMS* emergency medical service

### Outcomes and airway management

**Fig. 3:**
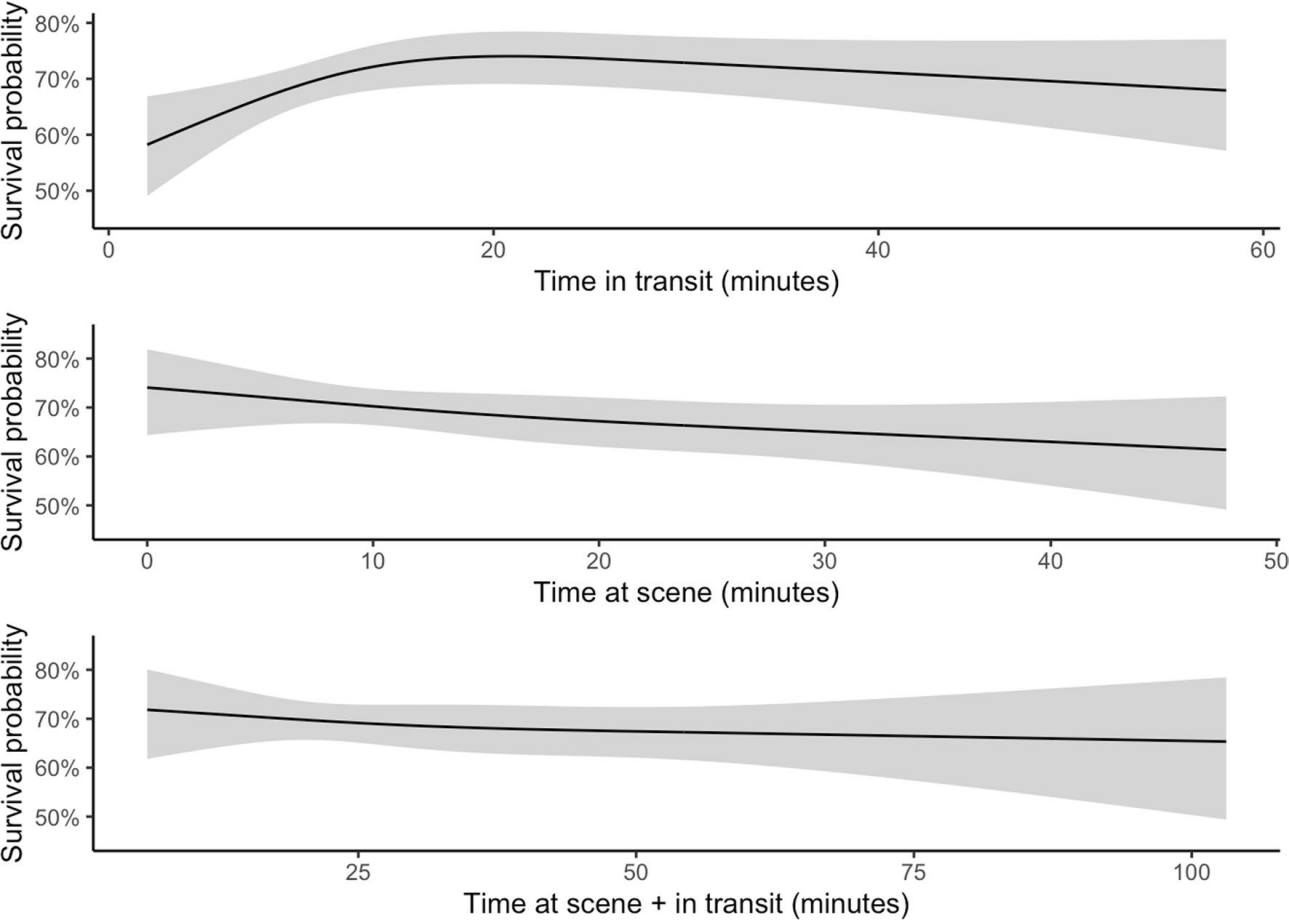
Mortality associated with transit time, scene time and combined scene and transit time for EMS

**Fig. 4:**
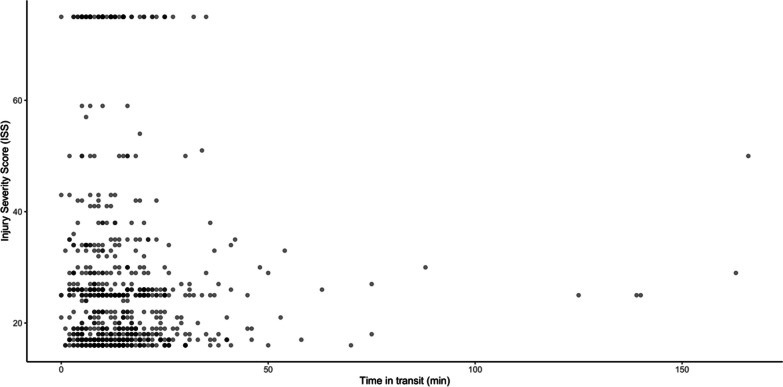
Injury severity score associated with transit time (minutes) for EMS. There was no association between ISS and transportation times. *ISS *injury severity score

The 30-day mortality for patients transported by EMS was 30% (n = 184), 50% (n = 5) for police transport, 22% (n = 6) for private vehicles, and all patients (n = 8) who arrived at the ED by foot survived (Table 3). Private vehicles, odds ratio (OR) 0.65 (confidence interval [CI] 0.24, 1.55, p = 0.4), and police transport, OR 2.28 (CI 0.63, 8.3, p = 0.2), were not associated with increased mortality in relation to EMS. The Glasgow outcome scale score was generally higher for patients transported by private vehicles and patients who arrived at the ED by foot compared with EMS and police transport. In total, 199 (32.5%) patients transported by EMS were intubated in the ED, compared with 6 (60%) patients transported by police and 12 (44.4%) patients transported by private vehicles. The mortality rates associated with transit time, scene time, and combined scene and transit time for EMS are presented in Fig. 3. Short transit times were significantly associated with increased mortality, but no other association was significant (Fig. 3). The ISS in relation to transport times for EMS is presented in Fig. 4.

**Table 3:** Outcomes

Characteristic	EMS, N = 612^a^	Police, N = 10^a^	Private vehicle, N = 27^a^
Ventilator days	1 (1, 3)	1 (1, 1)	2 (1, 5)
(Missing)	334	5	14
*30-day survival*			
Dead	184/612 (30%)	5/10 (50%)	6/27 (22%)
Alive	420/612 (69%)	5/10 (50%)	21/27 (78%)
Unknown	8/612 (1.3%)	0/10 (0%)	0/27 (0%)
*Glasgow outcome scale score*			
1	184/611 (30%)	5/10 (50%)	5/27 (19%)
2	5/611 (0.8%)	0/10 (0%)	1/27 (3.7%)
3	83/611 (14%)	1/10 (10%)	5/27 (19%)
4	200/611 (33%)	2/10 (20%)	4/27 (15%)
5	133/611 (22%)	2/10 (20%)	12/27 (44%)
Unknown	6/611 (1.0%)	0/10 (0%)	0/27 (0%)
(Missing)	1	0	0

^a^Median (IQR); n/N (%)

## Discussion

In this study, we showed that non-EMS transport of severe penetrating trauma occurred in 5.6% of cases. The mortality for police transport was 50% (n = 5) and 22% (n = 6) for private vehicles, and there was no mortality difference between EMS and police transport (OR 2.28 [CI 0.63, 8.3]) or private vehicles (OR 0.65 [CI 0.24, 1.55]). Adjusted mortality analysis of police transport and private vehicles was ceded due to limited sample size. The police transported 1.5% of the patients, who presented with lower GCS scores and a higher incidence of GSWs compared with EMS, in concurrence with earlier reports [8, 16, 17, 19, 20]. The combination of GSW and low GCS score may have signaled an urgency that prompted police to transport the victim instead of waiting for the EMS, although the specific reasons in these cases could not be deduced. In contrast to previous observations, ISS did not differ between patients transported by the police and EMS [8, 16, 18, 19]. Further analysis of ISS in relation to mode of transport showed that police transported patients with a lower ISS to a lesser extent than EMS, although the median ISS did not differ. The police transported more severely injured patients (median ISS 25) compared with earlier reports (mean ISS 14.2 and mean ISS 15.5) [8, 18], which is likely reflected in the increased mortality (50%) in relation to those reports (17.7% and 14.8%) [8, 18]. Private vehicles transported 4.1% of all cases, compared with previous observations of 12.6% and 20.5% [27, 28]. Patients transported by private vehicles had lower ISS, similar systolic blood pressure, and comparable GCS scores in relation to EMS, in concurrence with earlier reports [14, 27]. Private vehicles more frequently transported patients with GSW compared with EMS, which contrasts with a report from Wandling et al [20]. The median ISS 20 for patients transported by private vehicles was elevated in relation to earlier reports (median ISS 2 and 84% with mean ISS ≤ 15), which likely influenced the increased mortality (22%) compared with those reports (2.2% and 2.1%) [20, 27].

We detected a median scene time of 12 min for EMS. A nonsignificant trend of increased mortality with increased scene times was noted. Prehospital interventions may increase scene time and possible harm [3], and increased scene times have been associated with increased mortality [4, 29]. Advanced interventions enroute could lower the time on scene [30, 31]. Additionally, transport by non-EMS could decrease prehospital times [13, 15] and limit medical interventions. We found no association between ISS and transport time. In other studies, severely injured penetrating trauma patients were associated with shorter transport times [2], and shorter transport times increased mortality unrelated to injury severity [29]. These results may reflect an urgency in severely injured patients not necessarily mirrored in the present classification of injury severity.

The incidence of gun homicide in Philadelphia was 146 per million inhabitants in 2016. Several cities in the US have a similar incidence of gun homicide as Philadelphia without an established practice of police transports [18, 36], indicating additional contributing factors to the practice of non-EMS transport besides the incidence of gun homicide alone. In comparison, gun homicides occur at a rate of 4 per million inhabitants in Sweden and 1.6 per million inhabitants in Europe [1, 37]. Philadelphia has eight adult and pediatric trauma centers in proximity to shooting incidents, which is why conditions may be favorable for short transportation times by non-EMS [8, 28]. We have previously shown that the incidence of severe penetrating trauma was highest in the three largest metropolitan regions in Sweden [21]. These areas provide relatively short transportation times. Unsurprisingly, increased distance between the scene of violence and hospitals may increase mortality [32], and access to trauma centers in Sweden varies considerably depending on geographic location.[33] The availability of trauma centers within different healthcare organizations likely influences the challenges posed by prehospital triage. Accurate prehospital triage of trauma patients is challenging, and undertriage of undifferentiated trauma patients has been associated with increased mortality [34], with possible subsequent harm from interhospital transfers.[34, 35] Considering triage challenges by health care professionals, mistriage by non-EMS is likely elevated compared with EMS, with potential harmful effects on patients and health care resources.

The increased shooting incidence in Sweden also risks increasing the number of casualties in areas with ongoing violence, and anecdotal stories of police transport were discussed in Swedish media [38]. Here, we show that although transport by police and private vehicles occurred, the incidence was low. Nevertheless, in 2018, health and police authorities in the Stockholm region established an agreement that regulates the authorities’ cooperation concerning the management of severely injured patients around scenes of violence [39]. The agreement stated that EMS should always perform the transports unless time restraints or safety concerns dictate otherwise; in these circumstances, police may evacuate patients with a subsequent transfer to EMS at a safe location. Police transport to the hospital should be restricted to exceptional cases. Areas outside of Stockholm are still unregulated. Therefore, increased medical training of police officers may increase lifesaving interventions in either situation [40].

This study has some limitations that need to be acknowledged. First, this was an observational study with inherent limitations regarding association and causality. Second, prehospital deaths were not included in SweTrau, which may be a source of selection bias. Third, the number of non-EMS transports was small, which limited the analysis and decreased the observation confidence. Fourth, the coverage of SweTrau increased during the study period, which could affect outcomes, although we did not analyze trends.

## Conclusion

Non-EMS transport did occur, however with a low incidence and did not affect mortality. GSWs were more common in police transport, and victims had lower GCS scores when arriving at the ED, which warrants further investigations of the operational management of shooting victims in Sweden.

## Data Availability

The dataset analyzed during the current study is available in the SweTrau registry, [https://rcsyd.se/swetrau/].

## References

[1] Gun homicide in Sweden and other European countries. https://bra.se/download/18.1f8c9903175f8b2aa70ca53/1621930415477/2021_8_Gun_homicide_in_Sweden_and_other_European_countries.pdf: The Swedish National Council for Crime Prevention (Brå); 2021 (May 30, 2023).

[2] Swaroop M, Straus DC, Agubuzu O, Esposito TJ, Schermer CR, Crandall ML (2013). Pre-hospital transport times and survival for Hypotensive patients with penetrating thoracic trauma. J Emerg Trauma Shock.

[3] Harmsen AM, Giannakopoulos GF, Moerbeek PR, Jansma EP, Bonjer HJ, Bloemers FW (2015). The influence of prehospital time on trauma patients outcome: a systematic review. Injury.

[4] Brown JB, Rosengart MR, Forsythe RM, Reynolds BR, Gestring ML, Hallinan WM (2016). Not all prehospital time is equal: Influence of scene time on mortality. J Trauma Acute Care Surg.

[5] Seamon MJ, Fisher CA, Gaughan J, Lloyd M, Bradley KM, Santora TA (2007). Prehospital procedures before emergency department thoracotomy: "scoop and run" saves lives. J Trauma.

[6] Rappold JF, Hollenbach KA, Santora TA, Beadle D, Dauer ED, Sjoholm LO (2015). The evil of good is better: making the case for basic life support transport for penetrating trauma victims in an urban environment. J Trauma Acute Care Surg.

[7] Ninokawa S, Friedman J, Tatum D, Smith A, Taghavi S, McGrew P (2020). Patient contact time and prehospital interventions in hypotensive trauma patients: should we reconsider the "ABC" algorithm when time is of the essence?. Am Surg.

[8] Taghavi S, Maher Z, Goldberg AJ, Haut ER, Raza S, Chang G (2022). An analysis of police transport in an Eastern Association for the Surgery of Trauma multicenter trial examining prehospital procedures in penetrating trauma patients. J Trauma Acute Care Surg.

[9] Hudson AJ, Strandenes G, Bjerkvig CK, Svanevik M, Glassberg E (2018). Airway and ventilation management strategies for hemorrhagic shock. To tube, or not to tube, that is the question. J Trauma Acute Care Surg.

[10] Sakran JV, Mehta A, Fransman R, Nathens AB, Joseph B, Kent A (2018). Nationwide trends in mortality following penetrating trauma: Are we up for the challenge?. J Trauma Acute Care Surg.

[11] Pfeifer R, Halvachizadeh S, Schick S, Sprengel K, Jensen KO, Teuben M (2019). Are prehospital trauma deaths preventable? A systematic literature review. World J Surg.

[12] Jacoby SF, Reeping PM, Branas CC. Police-to Hospital Transport for Violently Injured Individuals: A Way to Save Lives? The ANNALS of the American Academy of Political and Social Science; 2020. pp. 186–201.

[13] Winter E, Byrne JP, Hynes AM, Geng Z, Seamon MJ, Holena DN (2022). Coming in hot: Police transport and prehospital time after firearm injury. J Trauma Acute Care Surg.

[14] Johnson NJ, Carr BG, Salhi R, Holena DN, Wolff C, Band RA (2013). Characteristics and outcomes of injured patients presenting by private vehicle in a state trauma system. Am J Emerg Med.

[15] Nasser AAH, Khouli Y (2020). The impact of prehospital transport mode on mortality of penetrating trauma patients. Air Med J.

[16] Winter E, Hynes AM, Shultz K, Holena DN, Malhotra NR, Cannon JW (2021). Association of police transport with survival among patients with penetrating trauma in Philadelphia, Pennsylvania. JAMA Netw Open.

[17] Band RA, Salhi RA, Holena DN, Powell E, Branas CC, Carr BG (2014). Severity-adjusted mortality in trauma patients transported by police. Ann Emerg Med.

[18] Wandling MW, Nathens AB, Shapiro MB, Haut ER (2016). Police transport versus ground EMS: a trauma system-level evaluation of prehospital care policies and their effect on clinical outcomes. J Trauma Acute Care Surg.

[19] Band RA, Pryor JP, Gaieski DF, Dickinson ET, Cummings D, Carr BG (2011). Injury-adjusted mortality of patients transported by police following penetrating trauma. Acad Emerg Med.

[20] Wandling MW, Nathens AB, Shapiro MB, Haut ER (2018). Association of prehospital mode of transport with mortality in penetrating trauma: a trauma system-level assessment of private vehicle transportation vs ground emergency medical services. JAMA Surg.

[21] Günther M, Dahlberg M, Rostami A, Azadali A, Arborelius UP, Linder F (2021). Incidence, demographics, and outcomes of penetrating trauma in Sweden during the past decade. Front Neurol.

[22] David JS, Bouzat P, Raux M (2019). Evolution and organisation of trauma systems. Anaesth Crit Care Pain Med..

[23] Beuran M, Paun S, Gaspar B, Vartic N, Hostiuc S, Chiotoroiu A (2012). Prehospital trauma care: a clinical review. Chirurgia (Bucur).

[24] Timmermann A, Russo SG, Hollmann MW (2008). Paramedic versus emergency physician emergency medical service: role of the anaesthesiologist and the European versus the Anglo-American concept. Curr Opin Anaesthesiol.

[25] Anual Report from the Swedish Trauma Registry (SweTrau) 2019. 2020.

[26] Brohi K (2008). The Utstein template for uniform reporting of data following major trauma: a valuable tool for establishing a pan-European dataset. Scand J Trauma Resusc Emerg Med..

[27] Zafar SN, Haider AH, Stevens KA, Ray-Mazumder N, Kisat MT, Schneider EB (2014). Increased mortality associated with EMS transport of gunshot wound victims when compared to private vehicle transport. Injury.

[28] Jacoby SF, Branas CC, Holena DN, Kaufman EJ (2020). Beyond survival: the broader consequences of prehospital transport by police for penetrating trauma. Trauma Surg Acute Care Open..

[29] Ruelas OS, Tschautscher CF, Lohse CM, Sztajnkrycer MD (2018). Analysis of prehospital scene times and interventions on mortality outcomes in a national cohort of penetrating and blunt trauma patients. Prehosp Emerg Care.

[30] Árnason B, Hertzberg D, Kornhall D, Günther M, Gellerfors M (2021). Pre-hospital emergency anaesthesia in trauma patients treated by anaesthesiologist and nurse anaesthetist staffed critical care teams. Acta Anaesthesiol Scand.

[31] Meizoso JP, Valle EJ, Allen CJ, Ray JJ, Jouria JM, Teisch LF (2015). Decreased mortality after prehospital interventions in severely injured trauma patients. J Trauma Acute Care Surg.

[32] Crandall M, Sharp D, Unger E, Straus D, Brasel K, Hsia R (2013). Trauma deserts: distance from a trauma center, transport times, and mortality from gunshot wounds in Chicago. Am J Public Health.

[33] Candefjord S, Asker L, Caragounis EC (2022). Mortality of trauma patients treated at trauma centers compared to nontrauma centers in Sweden: a retrospective study. Eur J Trauma Emerg Surg.

[34] Lupton JR, Davis-O'Reilly C, Jungbauer RM, Newgard CD, Fallat ME, Brown JB (2023). Under-triage and over-triage using the field triage guidelines for injured patients: a systematic review. Prehosp Emerg Care.

[35] Mans S, Reinders Folmer E, de Jongh MA, Lansink KW (2016). Direct transport versus inter hospital transfer of severely injured trauma patients. Injury.

[36] Houghton A, Jackson-Weaver O, Toraih E, Burley N, Byrne T, McGrew P (2021). Firearm homicide mortality is influenced by structural racism in US metropolitan areas. J Trauma Acute Care Surg.

[37] Philadelphia police department. Annual Murder and Shooting Victim Report:2016 https://www.phillypolice.com/assets/crime-maps-stats/2016-Homicide-Report.pdf2016.Accessed on May 27, 2023.

[38] Majlard J. Polis tvingas agera ambulans i hotfulla miljöer [Police are forced to act as ambulance in threatening environments]. Svenska dagbladet. 2018.

[39] Överenskommelse mellan ambulanssjukvården inom Stockholms läns landsting och polisen i region Stockholm avseende samarbete kring allvarligt skadade i pågående händelser med inslag av hot och våld. [Agreement between the ambulance medical service within the Stockholm County Council and the police in the Stockholm region regarding cooperation regarding seriously injured people in ongoing events with elements of threats and violence]. Polismyndigheten & Stockholms läns landsting [ Swedish Police & Stockholm County Council](2018).

[40] Wallin K, Holmberg M, Andersson H, Kronkvist O, Svensson A. The Emergency Care Competence Needed for Police Patrol Officers According to the Experts - a National Swedish Delphi Study. Nordic Journal of Studies in Policing; 2022. p. 1–15.

